# Inhaled Ciprofloxacin as an Alternative Treatment for Infection with *Coxiella burnetii*

**DOI:** 10.3390/antibiotics15030293

**Published:** 2026-03-13

**Authors:** Rachel E. Ireland, Kevin R. Bewley, M. Gill Hartley, Karleigh A. Hamblin, Stuart J. Armstrong, Michelle Nelson, Thomas R. Laws, Isobel H. Norville, Francisco J. Salguero, James D. Blanchard, Francis Dayton, Igor Gonda, Helen S. Atkins, Sarah V. Harding

**Affiliations:** 1 Defence Science and Technology Laboratory, Porton Down, Salisbury SP4 0JQ, UK; 2 UK Health Security Agency, Porton Down, Salisbury SP4 0JG, UK; kevin.bewley@ukhsa.gov.uk (K.R.B.);; 3 Aradigm Corporation, Hayward, CA 94545, USA; 4 Department of Respiratory Sciences, University of Leicester, Leicester LE1 7RH, UK

**Keywords:** Q fever, inhaled antibiotics, respiratory infection, mouse model, antimicrobial therapy

## Abstract

Background/Objectives: Q fever, caused by *Coxiella burnetii*, is typically treated with doxycycline, but its efficacy is limited in chronic cases and may be poorly tolerated. Systemic ciprofloxacin shows limited activity for acute Q fever. However, inhaled liposomal formulations may provide therapeutic benefit. Methods: Two inhaled ciprofloxacin formulations (Lipoquin^®^ and Apulmiq^®^) were evaluated in an A/J mouse model of Q fever and compared with intraperitoneal ciprofloxacin and oral doxycycline. Initially, pharmacokinetic studies were performed to determine an appropriate dosing regimen for the inhaled ciprofloxacin formulations. A separate cohort of mice were then infected with *C. burnetii* and treated once daily via nebulisation with Lipoquin or Apulmiq, initiated at 24, 48, or 72 h post-challenge. Clinical signs, weight change, splenomegaly, bacterial burden, and lung histopathology were evaluated. Results: Pharmacokinetic analysis confirmed sustained lung concentrations of inhaled ciprofloxacin, supporting once-daily dosing. Inhaled Lipoquin and Apulmiq significantly reduced clinical signs, weight loss, splenomegaly, and pulmonary bacterial burden compared to untreated controls and doxycycline-treated mice. Histopathology revealed decreased lung inflammation and lesion severity following inhalational dosing. Systemic ciprofloxacin slightly reduced splenic bacterial burden but was less effective in controlling pulmonary infection. Conclusions: Inhaled liposomal ciprofloxacin demonstrated superior protection and reduced respiratory manifestations of Q fever compared to doxycycline and systemic ciprofloxacin. These findings suggest inhaled formulations may represent a viable alternative for the treatment of Q fever pneumonia. Further studies are needed to evaluate clinical applicability and long-term outcomes.

## 1. Introduction

*Coxiella burnetii*, the causative agent of the zoonotic disease Q fever, is a Gram-negative bacterium that invades and replicates in the phagolysosome of host cells [1]. It has a very low infectious dose (1–10 organisms) and is transmitted predominantly by the aerosol route, infecting both animals and humans [2]. Q fever can manifest as a range of conditions, including asymptomatic disease, acute ‘flu-like’ disease, (including fever and headaches), or severe disease resulting in hospitalisation with conditions including hepatitis and pneumonia [3,4]. Typically, the disease is self-limiting, but as many as five percent of patients develop chronic Q fever, typically endocarditis or vascular infection. These infections are difficult to treat and can be fatal if untreated [5,6].

The tetracycline doxycycline is currently the most effective treatment for acute Q fever, typically resolving fever 2 to 3 days following initiation [4]. Alternative treatments including the macrolides, rifampicin and co-trimoxazole, are not as effective as doxycycline [7,8]. Chronic Q fever is harder to treat, with up to a 2-year regimen of doxycycline and hydroxychloroquine. Alternatively, treatment with a tetracycline plus a quinolone has been proposed more recently, if the tetracycline/hydroxychloroquine combination is not well tolerated [5,8]. Ciprofloxacin has been used successfully to treat endocarditis as a consequence of systemic infection with *C. burnetii* [9]. However, there is no evidence indicating that orally administered or injected ciprofloxacin would be an efficacious treatment for acute Q fever.

Novel therapies for the treatment of Q fever are warranted and are being investigated both in vitro and in vivo. These include the fluoroquinolones levofloxacin, moxifloxacin and finafloxacin, in addition to liposome-encapsulated ciprofloxacin, the latter delivered by the inhalational route to treat respiratory infections [10,11,12]. Inhaled antibiotics have been used for many years for the treatment of respiratory infections in patients with chronic lung conditions, including asthma, cystic fibrosis, and non-cystic fibrosis bronchiectasis (NCFB) [13]. The antibiotics that have been formulated for delivery by this route include the aminoglycosides amikacin, gentamicin, and tobramycin, as well as the fluoroquinolone ciprofloxacin [14,15].

Studies have shown that it is difficult to treat respiratory infections and pneumonia (typically caused by *Pseudomonas aeruginosa*) with intravenously delivered ciprofloxacin. This is due to the high concentrations of antibiotic required to reach the lungs to eradicate the colonising bacteria [16,17]. High doses have proved ineffective and have resulted in the development of resistance. The advantages of treating using inhaled antibiotics include (1) the prevention of bacterial dissemination (as the infection is treated whilst within the lung), potentially reducing the severity of disease; (2) the delivery of higher concentrations of antibiotic to the primary site of infection; and (3) the reduction in adverse effects associated with systemic administration, increasing tolerability and potentially increasing compliance [15,18].

Lipoquin^®^ and Apulmiq^®^ are formulations of ciprofloxacin that have been developed for delivery by the inhalation route using a nebuliser [19]. Lipoquin is composed of ciprofloxacin hydrochloride with >99% of the ciprofloxacin encapsulated in liposomes. Apulmiq is a 1:1 volume-to-volume mix of Lipoquin and a non-liposomal solution of ciprofloxacin. Both formulations have been evaluated for safety and tolerability, in human clinical trials [20]. The positive data reported is likely to be due to the encapsulation of ciprofloxacin in materials that are similar to human lung surfactant. The combination of the encapsulated ciprofloxacin and direct delivery to the lungs makes this treatment different from other formulations of ciprofloxacin. This results in good drug distribution and improved pharmacokinetics. The Apulmiq formulation enables a high initial concentration of ciprofloxacin (>1000-fold more than oral or injected ciprofloxacin) to be achieved from the “free” ciprofloxacin component. This is followed by the modified release of ciprofloxacin from the liposomal component. The modified release results in a longer duration of action, as the drug remains in the body for longer, indicated by an increased half-life compared to non-encapsulated ciprofloxacin [15]. An increased half-life has also been demonstrated in a clinical trial, with NCFB patients having a half-life of approximately 10 h in the lungs [21]. The modified release not only achieves sustained drug concentrations in the lung for maximum antibacterial efficacy but also allows for convenient once-daily dosing. Lipoquin and Apulmiq have performed well in Phase 2 clinical trials [22], but there were mixed results when using Apulmiq in Phase 3 clinical trials to treat lung infections with *P. aeruginosa* in patients with NCFB. However, a recent meta-analysis concluded that it was efficacious [23]. Both formulations have demonstrated tolerability and efficacy [22,24].

Lipoquin and Apulmiq have demonstrated utility against infections with *Francisella tularensis* strain SCHU S4 and *Yersinia pestis* strain CO92 in murine models when delivered by the inhalational route [25,26]. In addition, mice treated with intranasally delivered Lipoquin were protected against weight loss and the development of clinical signs of infection, following an aerosol challenge with *C. burnetii* strain Nine Mile [12]. This manuscript details further investigations of the liposome-encapsulated ciprofloxacin formulations as alternatives therapies for treating infections with *C. burnetii* in the A/J mouse. In these studies, treatment was initiated at 24, 48 and 72 h following an inhalational challenge with *C. burnetii* and was continued for seven days. Clinical signs of disease (including changes to body weight) were monitored for 14 days post-challenge and the bacterial burden and pathology in key tissues were compared to those collected from doxycycline- and ciprofloxacin-treated mice.

## 2. Results

### 2.1. The PK of Lipoquin and Apulmiq

The concentration–time profile was determined following the administration of Lipoquin and Apulmiq by the inhalational route and the PK parameters were determined by non-compartmental analysis (Table 1). The AUCs for ciprofloxacin in the plasma and lungs following Lipoquin inhalation were 3.27 µg·h/mL and 1.00 µg·h/g, respectively. For Apulmiq, the plasma and lung ciprofloxacin AUCs following inhalation were 1.23 µg·h/mL and 0.28 µg·h/g, respectively. The delivered lung dose was calculated as 1.27 mg/kg for Lipoquin for a 20 min exposure and 0.32 mg/kg for Apulmiq for a 30 min exposure using Equation (1). The target lung dose of 1 mg/kg (approximately equivalent to the dosing target for humans) was not reached for Apulmiq. In order to achieve this, modelling was performed to assess the effect of increasing the duration of dosing to 45 min. This is the maximum feasible duration for inhalational dosing for ethical reasons. Assuming a linear delivery of dose with time, the expected lung dose delivered following a 45 min exposure was 0.48 mg/kg. This was selected despite it being less that the target lung dose of 1 mg/kg. Therefore, a dosing regimen of a 20 min exposure to Lipoquin and a 45 min exposure to Apulmiq was selected for the efficacy study. Previously established regimens for the systemic delivery of ciprofloxacin and doxycycline were used in this study for comparison [12].

### 2.2. Liposomal Ciprofloxacin Reduced Clinical Disease and Weight Loss

**Figure 1:**
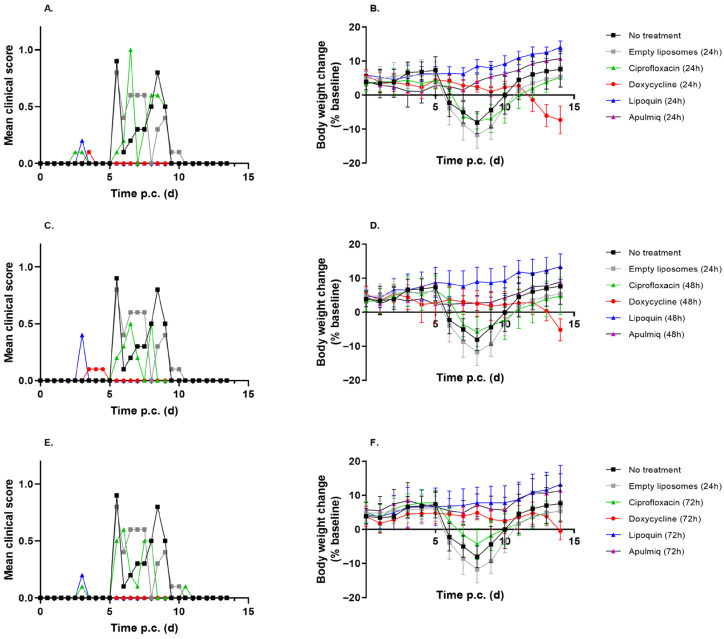
Clinical scores (**A**,**C**,**E**) and weight loss profiles (**B**,**D**,**F**) observed in mice following inhalational infection with *C. burnetii*.

The efficacy of Lipoquin and Apulmiq delivery by the inhalational route was compared to systemically delivered ciprofloxacin or the standard-of-care antibiotic for Q fever, doxycycline. Mice that did not receive treatment and those that received empty liposomes by the inhalational route developed clinical signs of disease. The clinical signs peaked on day 5 post-challenge and continued until day 10 post-challenge (Figure 1A). Body weight loss was also observed from day 6 post-challenge, with peak weight loss observed at day 8 post-challenge (Figure 1B). Interestingly, weight loss was greater in mice that received the empty liposomes compared to the untreated controls (*p* = 0.042 at day 7 post-challenge only). This may be due to the impact of the daily aerosol exposure. By day 8 post-challenge, these mice had regained this weight, and by the end of the study they had reached their pre-challenge weight.

Treatment with the liposomal ciprofloxacin formulations resulted in a reduction in the severity of disease when administered at 24, 48 and 72 h post-challenge. Only minor clinical signs were observed in mice treated with Lipoquin between days 3 and 5 post-challenge. No clinical signs were reported in mice treated with Apulmiq (Figure 1A,C,E). Both formulations provided significant protection from weight loss when administered at 24, 48 and 72 h post-challenge when compared to animals given empty liposome or left untreated (Figure 1B,D,F; *p* < 0.05, all cases).

In contrast, systemically delivered ciprofloxacin was less effective and mice presented with similar clinical signs to untreated or empty liposome-treated mice over a comparable period (Figure 1A,C,E). There was no protection from weight loss in mice that received ciprofloxacin compared to the untreated controls (*p* > 0.10), and the pattern of weight loss was delayed by one day when ciprofloxacin treatment commenced at 24 or 48 h post-challenge (Figure 1B,D). There was no delay in body weight loss in mice that were treated with ciprofloxacin at 72 h post-challenge (Figure 1F).

Mice that received oral doxycycline presented with minor clinical signs between days 3 and 5 post-challenge (Figure 1A,C,E). Doxycycline protected mice from weight loss on days 7 and 8 post-challenge when compared to untreated mice (Figure 1B,D,F; *p* < 0.05 for all groups). Mice maintained their body weight until day 11 post-challenge, after which weight loss occurred in the order corresponding to the time therapy was stopped, i.e., first in the animals that received antibiotics at 24 h, and then in the animals that received antibiotics at 48 and 72 h. This weight loss was comparable to that observed for the untreated controls and for mice that received systemically delivered ciprofloxacin.

Overall, weight loss was reduced when antibiotic treatment was initiated at 24 h post-challenge when compared to 72 h post-challenge (*p* = 0.008). Both Lipoquin and Apulmiq were more effective at preventing weight loss when compared to doxycycline and ciprofloxacin (*p* < 0.001), with Lipoquin proving more effective than Apulmiq (*p* = 0.008).

### 2.3. Liposomal Ciprofloxacin Reduced the Bacterial Burden in the Lungs

**Figure 2:**
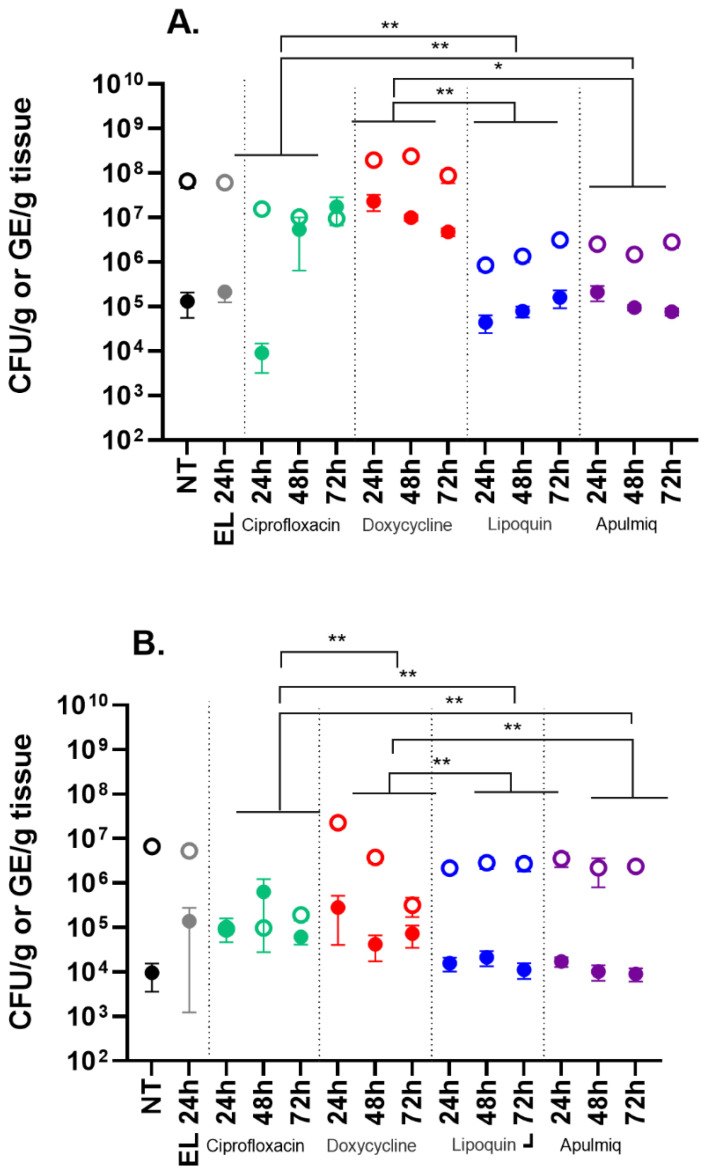
The bacterial burden in (**A**) lungs and (**B**) spleens of mice following inhalational infection with *C. burnetii*. Bacterial burden was assessed at the end of the study by qPCR (open circles) and CFU (closed circles). NT (black circle) no treatment, EL (grey circles) empty liposomes, green circles ciprofloxacin group, red circles doxycycline group, blue circles Lipoquin, purple circles apulmiq. Data are presented as the mean score and SEM for each group. Bacterial burden, determined by qPCR, data was analysed using a multivariate general linear statistical model with pairwise comparisons to assess the effect. * *p* < 0.05 and ** *p* < 0.01.

The bacterial burden in the lungs and spleen at the end of the study was determined using two different methods: we used qPCR to quantify the GE/mL and the enumeration of viable bacteria on solid ACCM2 media. High bacterial loads were recovered from all tissue samples (Figure 2). The bacterial burden determined by qPCR was analysed using a multivariate general linear statistical model to assess the effect of treatment and time of initiation of treatment. As expected, there was a treatment effect on the bacterial burden in both the lung and spleen, which was likely due to the time the treatment was initiated (*p* < 0.001). To consider differences between treatments, independent pairwise comparisons were performed. There was evidence for a reduction in the bacterial load in the lungs of mice receiving Lipoquin or Apulmiq, compared to systemically delivered ciprofloxacin (*p* < 0.006 for both, *n* = 30 per treatment) or to doxycycline (*p* < 0.006 and *p* = 0.018 respectively, *n* = 30 per treatment). The bacterial loads were also reduced in the spleens of mice receiving Lipoquin or Apulmiq compared to systemically delivered doxycycline (*p* < 0.006 for both, *n* = 30 per treatment). However, treatment with systemically delivered ciprofloxacin reduced the bacterial load in the spleens more effectively compared to Lipoquin or Apulmiq (*p* < 0.006 for both, *n* = 30 per treatment). Additionally, the bacterial burdens were reduced in the lungs and spleens of mice receiving systemically delivered ciprofloxacin compared to doxycycline (*p* < 0.006 for both, *n* = 30 per treatment). There was no evidence for differences in the bacterial load in mice that received Lipoquin or Apulmiq in the lungs (*p* > 0.108 for both, *n* = 30 per treatment) or spleen (*p* > 0.604 for both, *n* = 30 per treatment).

Samples were less available for the second method of quantification (enumerating viable *C. burnetii* on solid agar) as sections of the organs from a proportion of mice were prioritised for histopathological analyses. The analyses of the CFU data from the lung and spleen was therefore less powerful. There was evidence of an effect of treatment on the bacterial burden in the lungs compared to those harvested from the untreated controls which was also influenced by the time the treatment was initiated (*p* = 0.013, *n* = 6 per condition). Pairwise comparisons were performed to consider the differences between each treatment. There was no evidence of differences in the bacterial burdens in the lungs of mice receiving Lipoquin, compared to systemically delivered ciprofloxacin (*p* > 0.999, *n* = 18 per treatment). There was a reduction in the bacterial burdens in the lungs of mice receiving Apulmiq compared to systemically delivered ciprofloxacin when treatment was initiated at 48 and 72 h post-challenge (*p* = 0.03; *n* = 18 per treatment), but not at 24 h post-challenge. There was also evidence for a reduction in the bacterial load in the lungs of mice receiving Lipoquin or Apulmiq compared to systemically delivered doxycycline (*p* < 0.006 in both cases, *n* = 18 per treatment). When systemically delivered, ciprofloxacin was compared to doxycycline. There was evidence for a reduction in the bacterial burden in the lungs when treatment was initiated at 48 and 72 h post-challenge (*p* = 0.03; *n* = 18 per treatment), but not at 24 h post-challenge. There was no evidence for differences in the bacterial burden in the lungs of mice receiving Lipoquin when compared to Apulmiq (*p* > 0.504; *n* = 18 per treatment). There was also no evidence for an effect of treatment on the bacterial burden in the spleen (*p* = 0.571, *n* = 18 per treatment) or an effect of when treatment was initiated.

### 2.4. Liposomal Ciprofloxacin Controlled the Increase in Tissue Weight in Tissues

**Figure 3:**
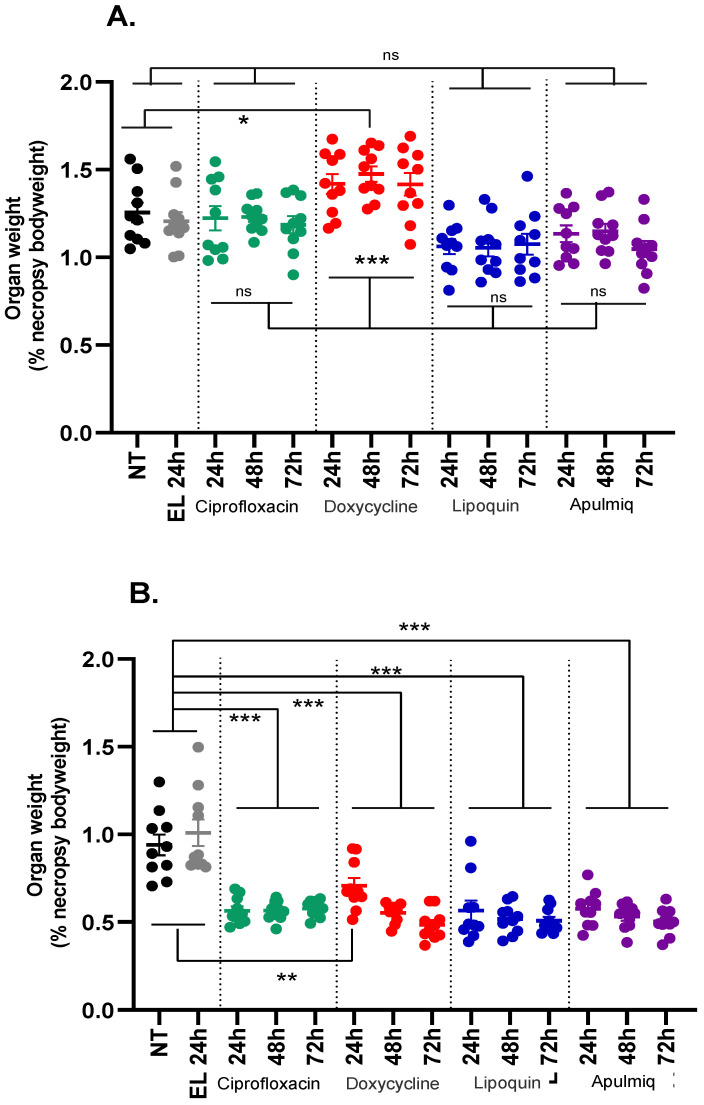
The organ weights of (**A**) lungs and (**B**) spleens at post-mortem in mice following inhalational infection with *C. burnetii*. NT (black circle) no treatment, EL (grey circles) empty liposomes, green circles ciprofloxacin group, red circles doxycycline group, blue circles Lipoquin, purple circles apulmiq. Data are presented as organ weight as a percentage of total body weight at necropsy. Organ weight data was analysed using a multivariate general linear statistical model with pairwise comparisons to assess the effect of treatment and time of initiation of treatment, where ns—not significant, * *p* < 0.05, ** *p* < 0.01 and *** *p* < 0.001.

Following the cessation of the study, all mice were euthanised and the spleens and lungs were weighed and collected to characterise general inflammation (Figure 3). Organ weight data was analysed using a multivariate general linear statistical model to assess the effect of treatment and the time of treatment initiation. Mice that had received ciprofloxacin systemically or the liposomal ciprofloxacin formulations by the inhalational route had lighter lungs than mice that received empty liposomes or were infected and untreated (*p* < 0.001, *n* = 30 per treatment), irrespective of when the treatment was initiated (*p* = 0.372, *n* = 30 per initiation time; Figure 3A). Mice that received doxycycline had heavier lungs when compared to mice that received empty liposomes or were infected and untreated (*p* < 0.001, *n* = 30 per treatment). This occurred irrespective of when the treatment was initiated (*p* = 0.372, *n* = 30 per initiation time). Simple pairwise comparisons of lung weights between treatment groups were performed and provided evidence for differences between all four treatment groups (*p* < 0.001, *n* = 30 per treatment). The exceptions were lungs from mice treated with ciprofloxacin when compared to those from mice treated with Apulmiq (*p* = 0.075, *n* = 30 per treatment) and when the lungs from mice treated with Lipoquin and Apulmiq were compared (*p* > 0.999, *n* = 30 per treatment). A positive effect on the spleen weight was also observed in mice that had received treatment, who had lighter spleens compared to mice that received empty liposomes or were infected and untreated. This was also influenced by the time of initiation of treatment (*p* = 0.013, *n* = 10 per combination of time and treatment; Figure 3B).

To further characterise the effects of time of treatment initiation and treatment on spleen weight, individual pairwise analyses were performed. There was a positive relationship between treatment type and the time of treatment initiation in the spleen weights harvested from mice treated with ciprofloxacin compared to those treated with doxycycline (*p* < 0.006, *n* = 10 per discrete combination of time and treatment).

### 2.5. Liposomal Ciprofloxacin Reduced the Severity of Pathology in Tissues

**Figure 4:**
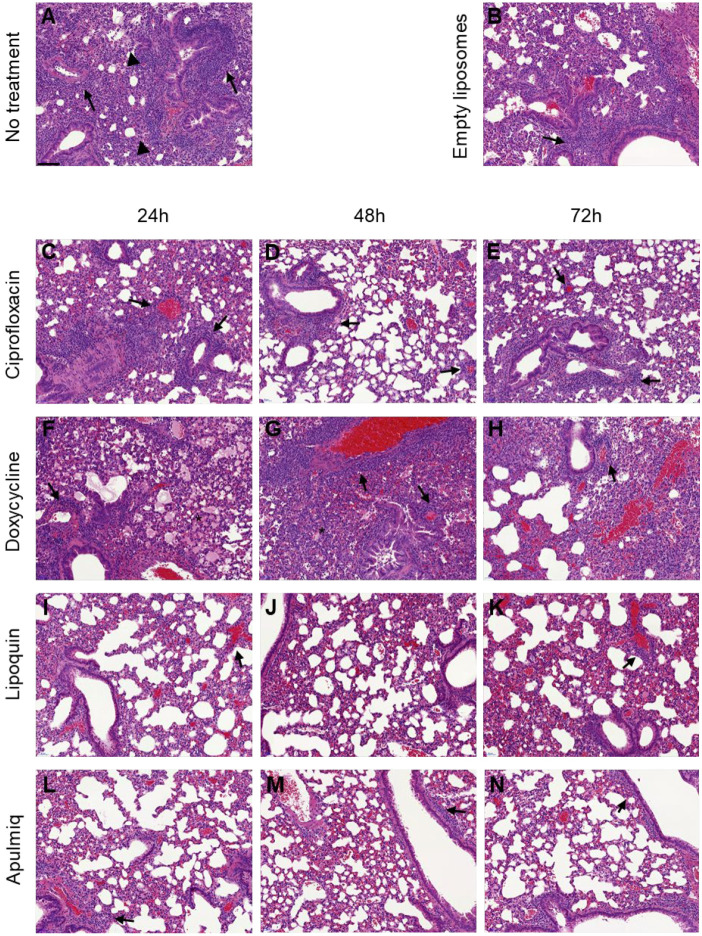
Representative H&E images from the lungs of mice following inhalational infection with *C. burnetii*. Perivascular and peribronchiolar cuffing (arrows) was observed in most animals, with reduced severity in animals treated with Lipoquin or Apulmiq. Acute focal bronchiolitis or alveolitis (*) was observed in animals treated with ciprofloxacin. Granulomatous alveolitis (arrowheads) was most commonly observed in untreated animals. Bar = 100 µm (in the untreated image).

Histopathological analysis was performed on the lungs and spleens of animals at the end of the study to assess disease severity. Perivascular and peribronchiolar cuffing was observed in most animals, with reduced severity in animals treated with Lipoquin or Apulmiq (Figure 4). There was also evidence that these treatments reduced the severity of vasculitis in the lungs. Acute focal bronchiolitis or alveolitis were also observed in animals treated with ciprofloxacin (Figure 4C–E). Acute lung lesions, bronchiolitis and alveolitis were observed in mice treated with doxycycline (Figure 4F–H). Granulomatous alveolitis was most commonly observed in untreated animals (Figure 4A,B).

**Figure 5:**
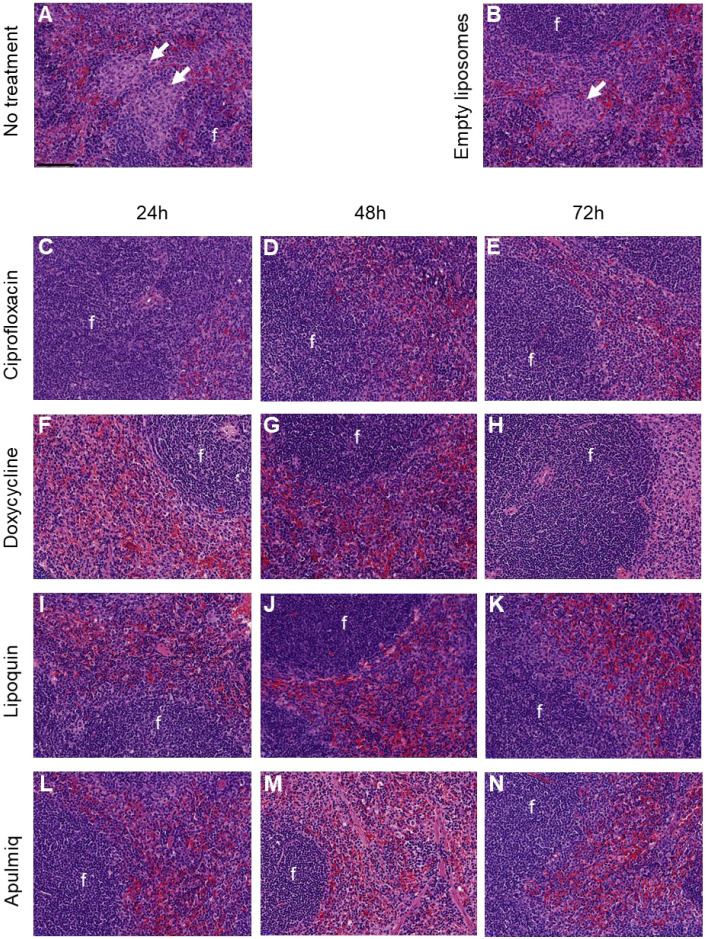
Representative H&E images from the spleens of mice following inhalational infection with *C. burnetii.* Multifocal granulomatous splenitis was observed in untreated animals or those treated with empty liposomes (arrows). These lesions were frequently observed in the red pulp, while the lymphoid follicles (f) did not show any considerable change. Bar = 100 µm (in the untreated image).

Focal granulomatous splenitis was observed in mice that did not receive treatment (Figure 5A). Multifocal granulomatous splenitis (ranging from normal to mild in severity) was observed in untreated mice and those that received empty liposomes (Figure 5A,B). These lesions were frequently observed in the red pulp, while the lymphoid follicles did not show any considerable change. Lesion severity was minimal or absent in mice that received antibiotic treatment (Figure 5C–N).

**Figure 6:**
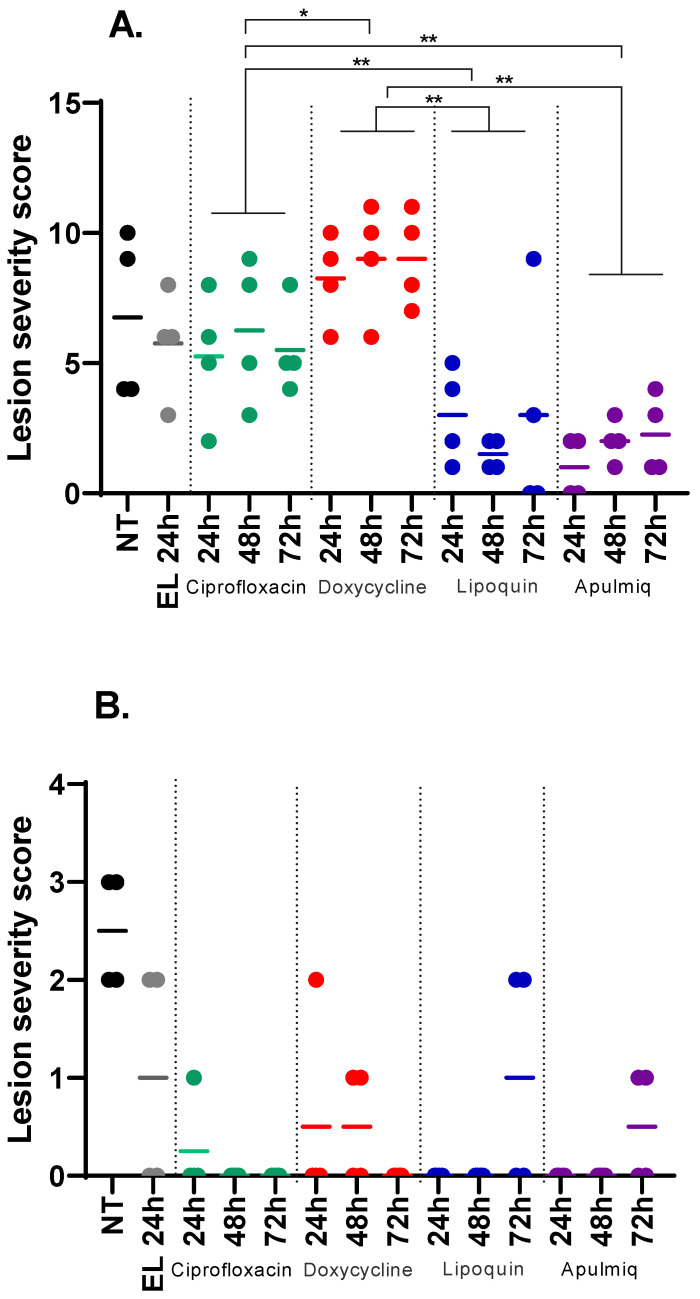
Lesion severity scores for the lungs (**A**) and spleens (**B**) of mice following inhalational infection with *C. burnetii*. NT (black circle) no treatment, EL (grey circles) empty liposomes, green circles ciprofloxacin group, red circles doxycycline group, blue circles Lipoquin, purple circles apulmiq. Lesion severity scores were analysed using an ordinal statistical model to assess overall differences and using the model’s coefficients to compare treatments, where * *p* < 0.05 and ** *p* < 0.01.

Lesion severity observed in tissues was evaluated using a scoring system (Figure 6).

Using an ordinal statistical model, there was evidence for overall differences in lesion severity in the lungs of mice that received antibiotic treatment compared to mice that were untreated or received empty liposomes (*p* < 0.001; Figure 6A). This was not the case when lesion severity was quantified in the spleen (*p* > 0.999; Figure 6B). The different treatments were compared using model coefficients. There was no evidence for differences in lung lesion severity in mice that received Apulmiq when compared to mice that received Lipoquin (*p* > 0.999; Figure 6A); however, lung lesion severity was reduced in mice treated with Lipoquin or Apulmiq when compared to mice that were treated with doxycycline or ciprofloxacin (*p* < 0.006 in all cases). Additionally, there was evidence for differences in lung lesion severity between the doxycycline-treated mice and ciprofloxacin-treated mice (*p* = 0.018). There was no evidence to indicate that the treatment initiation time had an effect on lung lesion severity (*p* = 0.680). There was also no evidence for differences in the effect of treatment (*p* = 0.250) or the treatment initiation time (*p* = 0.255) on spleen lesion severity (Figure 6B).

## 3. Discussion

Alternative therapies to oral doxycycline for the treatment of acute Q fever are required, primarily as there are issues with doxycycline intolerance [27]. People infected with acute Q fever may later develop chronic disease, even after effective treatment [6]. Chronic Q fever has a high mortality rate, potentially requiring further treatment and even surgical intervention. Inhaled antibiotics that can be self-administered (via an inhaler) would offer an advantage in reducing the burden on medical personnel and hospital facilities. In addition, directly delivering the antibiotic to the site of infection (lung) in an encapsulated form enables the slow, sustained release of higher concentrations of antibiotic and a reduction in the side effects associated with systemic administration [15,18].

The in vivo model of choice for these studies was the A/J mouse [12]. As with most of the human cases of Q fever, the infection in this model typically self-resolves, and so survival is not the primary parameter investigated [28]. The efficacy of therapies is determined by demonstrating differences in body weight, clinical signs of disease, the weight and bacterial load within organs and evidence of histopathological changes in the spleen and lung [29]. The antibiotic dosing regimen for all antibiotics was determined in the pharmacokinetic studies to ensure clinically relevant, human-equivalent doses were used for meaningful comparisons [12]. However, the target Apulmiq dose of 1 mg/kg could not be achieved. This value was based on human clinical trial data demonstrating efficacy in other infections and was therefore selected as the initial experimental dose for this study. One mg/kg dose was not achievable in the mice without exposing mice to the drug for upwards of 1.5 h a day. This was deemed not ethical, and so the duration of exposure was reduced. The fact that 0.48 mg/kg was efficacious highlights the non-linearity of scaling between humans and mice, which occurs due to various physiological differences, including faster metabolic rate, higher clearance rates, etc. That a lower dose in the mice was efficacious should mean that the greater dose delivered to humans is more likely to be beneficial.

The studies detailed here demonstrate differences between the treatments evaluated. Treatment with doxycycline resulted in a delay in bodyweight loss until treatment cessation. This has been observed previously and has been attributed to the bacteriostatic activity of doxycycline, keeping the infection under control until treatment is removed, when the disease becomes re-established [29]. As the standard doxycycline treatment is 14 days, this demonstrates the requirement for a well-tolerated treatment that will encourage patients to complete the course. Ciprofloxacin was ineffective against acute disease, likely due to the acidic environment of the parasitic vacuole where *C. burnetii* resides [1]. It has previously been reported that the activity of ciprofloxacin is reduced at low pH values [30,31]. Encapsulating ciprofloxacin within liposomes encourages phagocytic uptake by macrophages, possibly trafficking them to the parasitic vesicles, thereby bringing the bacterium and the antibiotic into closer proximity. There is also evidence that suggests that encapsulated ciprofloxacin can induce macrophages to become more classically activated, increasing their bacteriostatic activities, important for controlling *C. burnetii* growth [32,33].

Treatment with either Lipoquin or Apulmiq was more effective than doxycycline at controlling acute disease, preventing weight loss, and the development of clinical signs. This aligns with previously published data reporting protection against acute disease when Lipoquin was delivered by the intranasal route [12]. At the end of the study period, both Lipoquin and Apulmiq were also shown to have been more effective than doxycycline at preventing bacterial colonisation of the lung, as determined by a change in lung weight, the bacterial burden enumerated by PCR or viable counts, and by histological analysis. This was unaffected by treatment initiation time, although no clinical signs were observed in the animals. Bacterial clearance was not observed, although this is comparable to human disease, and could lead to chronic or recurrent infection. The clearance of *C. burnetii* following doxycycline treatment relies on a strong adaptive immune response, reducing the development of chronic disease [34]. Indeed, the best prognosis occurs where treatment is initiated several days after a known exposure to allow sufficient time for the stimulation of the adaptive immune system [35]. Treatment too early may prevent this complementary host response, although this time of initiation of treatment had a negligible effect in this study. The number of viable bacteria recovered from the lungs and spleens of animals at the end of the study was lower than the number recovered by qPCR, which may indicate ongoing bacterial killing.

The enhanced protection observed by liposomally encapsulated ciprofloxacin is related to the high encapsulation efficiency and the narrow size distribution (~100 nm) [15,19]. The laboratory determined that zeta potential in non-physiological media does not appear to be relevant to the conditions in vivo but is the same for Apulmiq and Lipoquin as the liposomal encapsulated formulation is the same. Although no difference in protection was observed in the Q fever mouse, protection from Apulmiq is generally superior in a number of animal models and human studies [21,24,36]. The unencapsulated ciprofloxacin in the Apulmiq formulation is rapidly absorbed from the respiratory surfaces without being phagocytosed or endocytosed and enters the bloodstream to treat the extracellular bacteria. However, the size and nature of the liposomes facilitate phagocytosis or endocytosis and result in the killing of intracellular bacteria. 

All of the treatments prevented splenomegaly and reduced pathology within the spleen, both signs of acute systemic disease [37,38]. Ciprofloxacin was marginally more successful at reducing systemic bacterial dissemination compared to what was observed in untreated animals. The relevance of systemic control is unclear, but *C. burnetii* has been shown to persist for long periods of time in sites of limited immune penetration [39,40]. It is possible that *C. burnetii* had already been trafficked away from the lungs within the first few hours of infection, prior to the initiation of the antibiotics, especially considering the high level of exposure required to initiate disease in the mouse model.

## 4. Materials and Methods

### 4.1. Bacteria

All work with *C. burnetii* was carried out within Class III microbiological safety cabinets within a ACDP Containment Level 3 (CL3) laboratory in facilities that are registered and inspected by the UK Health and Safety Executive, as required under the Control of Substances Hazardous to Health (COSHH) regulations and all associated guidance for CL3 pathogens. *C. burnetii* was handled in accordance with safety documentation that was reviewed by the Dstl Biological Safety Risk Committee and the Biological Safety Officer and also authorised by management.

*C. burnetii* strain Nine Mile was grown axenically in 100 mL acidified citrate cysteine medium 2 (ACCM-2) broth, incubated at 37 °C, and shaken at 75 rpm for 6 days, with a GENbox microaer atmosphere generator (bioMérieux, Basingstoke, Hampshire, UK) employed to displace oxygen. Bacteria were harvested by centrifugation at 10,000× *g* at 21 °C for 20 min and re-suspended in sterile PBS at a concentration of approximately 1 × 10^9^ genome equivalents (GE)/mL. Bacterial enumeration was determined by quantitative real-time qPCR (RT-qPCR), as described below, or by plating onto ACCM-2 agar plates and incubating at 37 °C for 14 days in 2.5%O_2_ 5% CO_2_.

### 4.2. A/Jola Mice

Animal studies were carried out in accordance with the UK Animals (Scientific Procedures) Act 1986 under a Home Office Project Licence and an Animal Care and Use Review Office (ACURO) Appendix. Groups of age-matched (minimum of 6 weeks old) male A/Jola (A/J) mice were obtained from Envigo (Belton, Leicestershire, UK). On receipt animals were randomised into cages using randomisation tables. No confounding factors were controlled for. Food and water were available ad libitum, and animals were housed within an ACDP Level 2 laboratory (for the pharmacokinetic studies) or an ACDP CL3 flexible-film isolator (for the efficacy studies) and allowed to acclimatise for a minimum of 5 days before exposure. Following challenge, animals were assessed twice a day for clinical signs. Clinical signs were determined as follows: a healthy animal was assigned 0; a ruffled and dehydrated animal was assigned 1; an animal with an arched back, eyes closed, and wasp-waisted appearance was assigned 2; immobility received a score of 9. As the disease is not lethal, no humane endpoint was applied, and no animals were euthanised based on clinical scores. Staff performing procedures and assessing the animals were not specifically blinded to the treatment.

### 4.3. Antibiotics

Apulmiq (35 mg/mL) was produced by mixing equal volumes of Lipoquin (50 mg/mL) and free ciprofloxacin solution (20 mg/mL). The Lipoquin or Apulmiq aerosols were generated using a Pari LC Star Sprint nebuliser powered by a Pari Boy SX compressor (Pari Medical Ltd. West, Byfleet, Surrey, UK). Mice received the inhaled antibiotics using an Inhalation Therapy System (ITS) [25] via the attached head-only exposure chamber. Ciprofloxacin (22 mg/kg) was administered by the i.p. and doxycycline (100 mg/kg) by the oral route twice daily.

### 4.4. Bacterial Enumeration of C. burnetii by Real-Time Quantitative (q)PCR

q-PCR targeted the *icd* gene of *C. burnetii* using the forward primer gttcccagccaaggtgaaaa and reverse primer gggtcggtcaggaacttctaaa. The sequence of the probe was atcaccgttaataaagc, which was covalently labelled at the 5′ end with the reporter dye 6-carboxyfluorescein (FAM) and at the 3′ end with a Minor Groove Binding (MGB) non-fluorescent quencher. Bacterial chromosomal DNA was extracted from animal tissues using the Qiagen QIAmp DNA minikit/blood mini/tissue kit, as per the manufacturer’s instructions. A typical RT-PCR mixture comprised 2 μL template DNA, forward primer (900 nM), reverse primer (900 nM), probe (250 nM), and ABI Fast TaqMan mastermix. PCR cycling conditions were 3 min at 95 °C, 30 s at 60 °C, followed by 50 two-step cycles, with one cycle consisting of 15 s at 95 °C and 30 s at 60 °C. For each PCR, a control of linearized synthetic plasmid containing a single copy of the target was included. The concentration of synthetic plasmid was quantified following linearisation and purification using an ND-2500 NanoDrop spectrophotometer. For each reaction, a standard curve of the synthetic plasmid was run in duplicate for the range of 1 × 10^7^ GE/mL to 1 × 10^2^ GE/mL. A plasmid concentration of 1 × 10^1^ GE/mL in triplicate was used as a lower-limit check in each assay. The limit of detection for this assay is 2.5 × 10^3^ GE/mL (bacterial culture), 4.4 × 10^1^ GE/mg (spleen), or 2.2 × 10^1^ GE/mg (lung).

### 4.5. Pharmacokinetics (PK) of Lipoquin and Apulmiq

Lipoquin (50 mg/mL in a 4 mL volume) or Apulmiq (20 mg/mL in a 6 mL volume) were administered to mice (*n* = 33 per treatment) by the inhalational route using the ITS described in the Section 4.3 section over a period of 20 or 30 min, respectively. The dose used for the PK analysis was the mean lung concentration at 0.017, h corrected for lung weight and normalised by bodyweight (18 g), as shown in Equation (1). Blood was collected via cardiac puncture under terminal anaesthesia into lithium heparin tubes from groups of 3 mice at 0.017, 0.25, 0.5, 1, 2, 4, 6 or 8 (for Lipoquin and Apulmiq, respectively), 10 or 12 (for Lipoquin and Apulmiq, respectively), 18, and 24 h post-dosing. The lungs were also collected from all animals, post-mortem, to quantify the concentrations of ciprofloxacin deposited (Equation (1)). It is acknowledged that not all of the inhaled dose will be retained or indeed reach the lungs, with a proportion of the inhaled dose reaching, e.g., the stomach. Therefore, this calculation was used for experimental purposes to assist with determining the dose required for the efficacy studies.

(1) $$Calculated\;deposited\;Dose\;(ng/kg)=\frac{\left(mean\;lung\;concentration\;at\;0.017h\;(ng/g)\times lung\;mass\;(g)\right)}{Mouse\;body\;mass\;(kg)}$$

Equation (1). Method used to calculate the dose of Lipoquin or Apulmiq delivered by the inhalational route measured post-dosing.

Blood samples were centrifuged to separate the plasma from the whole blood. The plasma and lungs were stored at −80 °C until analysis by high-performance liquid chromatography (HPLC) at Q3 Analytical (Porton Down, UK).

Non-compartmental pharmacokinetic analysis of the concentration–time data was completed using Phoenix WinNonlin (v8.3, Certara Inc., Radnor, PA, USA). Following the determination of the Lipoquin and Apulmiq concentration–time profiles in mice, the murine human-equivalent doses were calculated as the product of murine clearance and human AUCs (1.22 µg·h/mL and 0.92 µg·h/mL, for Lipoquin and Apulmiq respectively) [26] using *Equation (2)*. Data from all animals were included in the analysis.

(2) $${Dose}_{murine}={AUC}_{human}\times {Clearance}_{murine}$$

Equation (2). Method of calculating the human-equivalent dose of Lipoquin and Apulmiq, adapted from [41].

### 4.6. Antibiotic Efficacy in A/J Mice Infected with C. burnetii

The *C. burnetii* bacterial challenge was prepared as described earlier. Mice were challenged with an aerosol produced from a 10 mL suspension of *C. burnetii* at a concentration of approximately 1 × 10^9^ GE/mL using an AeroMP apparatus. The bacterial aerosol was generated using a six-jet Collison nebuliser (BGI, Waltham, MA, USA) operating at 15 L/min. The aerosol was mixed with conditioned air in the spray tube and delivered via a head-only exposure chamber. Samples of the aerosol were taken using an AGI-30 (Ace Glass Inc., Vineland, NJ, USA) at 6 L/min containing PBS, which was controlled and monitored using the AeroMP management platform (Biaera Technologies, LLC, Hagerstown, MD, USA). The back titration of the aerosol samples taken at the time of challenge was performed using RT-PCR, as described above. The retained dose of bacteria that mice received in each run was calculated by applying the Guyton formula [42]. It was assumed that each mouse retained 40% of the organisms that were inhaled [43].

Groups of 10 mice were treated twice daily with ciprofloxacin (22 mg/kg) by the intraperitoneal route (Ciproxin; Bayer UK, Reading, Berkshire, UK), twice daily with doxycycline by the oral route (100 mg/kg in sterile water: Vibramycin D; Pfizer, Tadworth, Surrey, UK), once daily with Lipoquin (Aradigm, Hayward, CA, USA) by the inhalational route (1 mg/kg lung dose delivered; 20 min nebulisation), or once daily with Apulmiq (Aradigm) by the inhalational route (0.48 mg/kg lung dose; 45 min nebulisation), with treatment initiated at 24, 48 or 72 h post-challenge. A control group of mice (*n* = 10) were treated once daily with empty liposomes (Aradigm) initiated at 24 h post-challenge (20 min nebulisation). Treatments were continued for 7 days. A challenged but untreated group was also included (*n* = 10).

Mice were observed for 14 days post-challenge, with individual animal weights recorded daily and clinical signs recorded twice daily. At the end of the study, the mice were humanely euthanised and the spleen and lungs weighed and collected for bacterial burden by colony count (*n* = 6) or RT-PCR (*n* = 10) and for histopathological analysis (*n* = 4). The experiment was performed as two separate studies (*n* = 5 per group) due to the logistics and capacity of the animal facility. The data from these replicate studies were combined (*n* = 10 per group) for statistical analysis. Data from all animals were used in the analysis.

### 4.7. Histopathology

Sections of the spleens and lungs (n = 4 per group) were fixed in 10% neutral buffered formalin, processed using paraffin wax, cut into 3–5 µm thick sections, and stained with hematoxylin and eosin (H&E). The slides were scanned with a Hamamatsu S360 scanner and analysed with ndpview2 software v2.9.22. Slides were randomised prior to examination to prevent bias and were evaluated subjectively using a scoring system to evaluate different parameters observed in the tissues. For the spleen samples, the presence of granulomatous splenitis with multifocal infiltrates of macrophages within both the red and white pulp (lymphoid follicles) was scored as follows: 0—within normal limits, 1—minimal, 2—mild, 3—moderate, and 4—marked/severe. The pathologist was blinded to the treatment group until the slides were scored.

For the lung, five parameters were evaluated: acute bronchiolitis (infiltration of airway walls and lumen by neutrophils), acute alveolitis (infiltration of alveolar walls and spaces by neutrophils), granulomatous alveolitis (infiltration of alveolar walls and spaces by macrophages and lymphocytes), perivascular and peribronchiolar cuffs (infiltration of lymphocytes into tissues around vessels and airways), and vasculitis (focal infiltration of blood vessel walls by lymphocytes, with variable margination of lymphocytes associated with the tunica intima). These parameters were also scored on a 0–4 basis, as described above. The pathologist was blinded to the treatment group until the slides were scored.

### 4.8. Statistical Analysis

Graphs were prepared using Prism (v8.0 GraphPad Inc., Boston, MA, USA) and analyses were performed using Prism or SPSS (v27.0 IBM). Body weight loss during the study was the primary measure and was compared to body weight at exposure using multivariate and repeated measures General Linear Models (GLM). Sphericity was not assumed; where required, the Greenhouse–Geisser correction was used. Bacterial burden data (CFU/mL and GE/mL) and organ weight data were first transformed by the natural logarithm to better fit the normal distribution, which was assessed using a quantile–quantile plot. Prior to transformation, 1 was added to the CFU to manage a small number of zero values. These data sets were analysed by multivariate analysis. During modelling, multiple models were run and a Bonferroni’s correction was manually applied. Histology score data was analysed using ordinal logistic generalised linear models. Two approaches were used: The first approach treated each group (treatment initiation time and treatment) as an independent group, with the untreated group used as the base level. In the second approach, forward model building was used to consider which parameters were important [44]. The inclusion criterion was set to *p* = 0.10 using a log likelihood test. Individual differences were compared using the same method with a Bonferroni’s correction.

## 5. Conclusions

This study demonstrates that the inhaled liposomal ciprofloxacin formulations Lipoquin and Apulmiq provided therapeutic benefit against acute Q fever, outperforming human-equivalent doses of both standard doxycycline therapy and systemic ciprofloxacin. Both inhaled formulations were similarly effective, with Apulmiq better improving clinical signs and Lipoquin slightly superior in limiting weight loss. Treatment at 24 h resulted in slightly better prevention of weight loss, as well as a slight reduction in organ pathology, although all initiation times gave good protection.

The key advantage of inhaled drugs is the direct pulmonary delivery, which achieves high, sustained antibiotic levels in the lung and supports convenient once-daily dosing. This is particularly important given the real-world challenges of doxycycline intolerance, contraindications, and adherence issues that can contribute to treatment failure and progression to chronic Q fever.

Despite promising efficacy, the study also notes limitations: complete bacterial clearance was not achieved, and the 14-day study duration did not allow the assessment of long-term persistence or the risk of chronic disease.

Overall, inhaled liposomal ciprofloxacin shows significant potential as a practical alternative to doxycycline for acute Q fever. Further studies are required to evaluate long-term outcomes, optimise dosing, and determine whether these formulations can ultimately help prevent chronic Q fever in patients.

## Figures and Tables

**Table 1:** Pharmacokinetic parameters of ciprofloxacin in mouse plasma and lung homogenates following a single dose of Lipoquin or Apulmiq delivered by the inhalational route. A/J mice (*n* = 33 per treatment) were exposed to Lipoquin (50 mg/mL in a 4 mL volume) or Apulmiq (20 mg/mL in a 6 mL volume) by the inhalational route using the ITS for 20 or 30 min, respectively. Blood and lungs were collected from animals at various timepoints and the concentration of ciprofloxacin was quantified by HPLC. Non-compartmental pharmacokinetic analysis of the concentration–time data was completed using Phoenix WinNonlin (v8.3, Certara Inc.).

Formulation	Lipoquin		Apulmiq	
**Measured lung dose**	1.27 mg/kg		0.32 mg/kg	
Parameter	Plasma	Lung	Plasma	Lung
R^2^ adjusted	0.924	0.994	0.922	0.971
Number of point used to estimate λ_z_	8	6	6	9
λ_z_	0.25 h^−1^	0.15 h^−1^	0.17 h^−1^	0.14 h^−1^
T½	2.76 h	4.57 h	3.99 h	4.83
C_max_	1.22 ± 0.12 μg/mL	1.38 μg/g	0.41 ± 0.15 μg/mL	35.3 μg/g
T_max_	0.25 h	Not applicable	1.0	n/a
AUC	3.27 ± 0.33 h·μg/mL	995.8 ± 68.8 h·ng/g	1.23 ± 0.14 h·μg/mL	274.6 ± 14.9 h·ng/g
CL	387 mL/h/kg	8.2 g/h/kg	255 mL/h/kg	1.1 g/h/kg
V	1539 mL/kg	1.24 g/kg	1469 mL/kg	7.76 g/kg
MRT	2.85 h	5.44 h	3.44 h	5.56 h

λ_z_ terminal elimination rate constant, T_1/2_ elimination half-life, C_max_ maximum concentration, AUC Area under the curve, CL total plasma clearance, V apparent volume of distribution, MRT mean residence time.

## Data Availability

The original contributions presented in this study are included in the article. Further inquiries can be directed to the corresponding author.
